# ﻿*Thliphthisasapphus* (Rubiaceae, Rubieae), a new species from Lefkada (Ionian Islands, Greece) and its ecological position

**DOI:** 10.3897/phytokeys.241.119144

**Published:** 2024-04-09

**Authors:** Walter Gutermann, Tae-Soo Jang, Arndt Kästner, David Prehsler, Dieter Reich, Andreas Berger, Ruth Flatscher, Christian Gilli, Markus Hofbauer, Margarita Lachmayer, Ruth Sander, Michaela Sonnleitner, Ladislav Mucina

**Affiliations:** 1 Division of Systematic and Evolutionary Botany, Department of Botany and Biodiversity Research, University of Vienna, Rennweg 14, Vienna, 1030, Austria; 2 Department of Biological Science, College of Bioscience and Biotechnology, Chungnam National University, Daejeon 34134, Republic of Korea; 3 Robert-Koch-Straße 29b, 06111 Halle (Saale), Germany; 4 Core Facility Botanical Garden, University of Vienna, Rennweg 14, Vienna, 1030, Austria; 5 Department of Botany, Natural History Museum Vienna, Burgring 7, Vienna, 1010, Austria; 6 Division of Structural and Functional Botany, Department of Botany and Biodiversity Research, University of Vienna, Rennweg 14, Vienna, 1030, Austria; 7 Harry Butler Institute, Murdoch University, 90 South Street, Building 390, Murdoch WA 6150, Perth, Australia; 8 Department of Geography & Environmental Studies, Stellenbosch University, Private Bag X1, Matieland 7602, Stellenbosch, South Africa; † Deceased

**Keywords:** *Asperula*, coastal cliff refugia, Greece, Ionian Islands, phytosociology, Rubiaceae, species nova, *Thliphthisa*

## Abstract

The new species, *Thliphthisasapphus***sp. nov.** (Rubiaceae, Rubieae), a narrow endemic of the white cliffs of Lefkátas on the southwest coast of Lefkada (Greece) is described and illustrated and an IUCN assessment is presented. Vegetation relevés were performed at the single known locality, limestone cliffs facing the sea and revealed a new association, the *Thliphthisasapphus*-*Lomelosietumdallaportae*. The chromosome number of *Thliphthisasapphus* was determined as 2*n* = 4*x* = 44, being the single tetraploid species in the genus to date. The species also differs markedly morphologically from its morphologically closest relatives, two Greek steno-endemic oreophytes, *Th.baenitzii* and *Th.muscosa* by the following characters: densely setose mericarps and corolla, tetraploidy and by its distribution. An identification key for the Greek species of *Thliphthisa* is provided. *Th.sapphus* constitutes the westernmost outpost of a group of Greek steno-endemics, highlighting the importance of coastal habitats and their protection as refugia for poorly competitive chamaephytes.

## ﻿Introduction

The recently erected genus *Thliphthisa* (Griseb.) P.Caputo & Del Guacchio (Rubiaceae, Rubieae, Galiinae) is centred in the eastern Mediterranean and includes 22 species, mostly narrow endemics. The systematics and taxonomy of subtribe Galiinae with up to 900 species is complex and the genus boundaries are still disputed, but the group is generally recognised by herbaceous habit with pseudo-whorled leaves and dry mericarps (Ehrendorfer et al. 2018). According to traditional understanding, the group is divided into several small (e.g. *Sherardia* L.) to medium-sized genera (e.g. *Asperula* L.) and the large, para- and polyphyletic genus *Galium* L. with about 650 species. As a result of extensive morphological and phylogenetic studies, Ehrendorfer and co-workers recently proposed that several sections of *Asperula* and *Galium* be elevated to generic rank, which would allow the retention of all traditional genera of Galiinae such as *Cruciata* Mill., *Sherardia* or *Valantia* L., a viewpoint favoured over merging the whole Galiinae resulting in a large and heterogeneous *Galium* (s. latiss.) (Ehrendorfer and Barfuss 2014; Ehrendorfer et al. 2018). Following these suggestions, Galiinae should comprise more than a dozen small genera, but most formal taxonomic decisions following these results are still pending.

*Asperula* sects. *Cynanchicae* (DC.) Boiss., *Thliphthisa* (Griseb.) Ehrend. and *Hexaphylla* Klokov were consistently resolved as monophyletic groups together with the monophyletic *Sherardia*, but the relationships of these groups are not always resolved (*Sherardia* clade of Natali et al. (1995); Clade IV of Soza and Olmstead (2010); Clade VI of Ehrendorfer et al. (2018)). Following Ehrendorfer and co-workers, Del Guacchio and Caputo (2020) recently proposed to raise these sections to generic rank basically upgrading the “distinguishable smaller subclades” of Clade VI (Ehrendorfer et al. 2018) to generic level. Morphologically, *Thliphthisa* is variable and difficult to separate from other representatives of subtribe Galiinae, one of the few consistent characters being truncate fruits (Schönbeck-Temesy and Ehrendorfer 1985; Del Guacchio and Caputo 2020). In *Thliphthisa*, Del Guacchio and Caputo (2020) have combined all but one taxon treated in the synopsis (conspectus, key to species and distribution maps) of Schönbeck-Temesy and Ehrendorfer (1985), although without citing this revision. Only the African *A.cyrenaica* (E.A.Durand & Barratte) Pamp. was included in genus *Hexaphylla* (Klokov) P.Caputo & Del Guacchio.

In November 1994, during floristic and vegetation studies on the island of Lefkada, Ionian Islands, Greece, a presumably undescribed rubiaceous chamaephyte was found. At the time of discovery, the plants had passed flowering and only remnants of withered corollas and aborted fruits were found. Repeated study of the population in summer 1996 as well as spring 2000 and 2011, observations on plants grown in cultivation and herbarium studies comparing all other Mediterranean species of *Asperula* s.l. confirmed that the enigmatic taxon is a *Thliphthisa* species new to science. It is here described as *Thliphthisasapphus*, its affinities, cytology and ecology are detailed and an identification key for the Greek species of the genus is provided.

## ﻿Materials and methods

Due to the steep cliff terrain, direct observations are limited to a small part of the single known population of this new *Thliphthisa* species. Vegetation relevés using a modified Braun-Blanquet cover-abundance scale (see Barkman et al. (1964) for details) have been made at the habitats of the species in autumn 1994 and in spring 1996. These relevés have been combined into a table which was further compared to known vegetation types of chasmophytic vegetation described so far from the Mediterranean to establish the syntaxonomic community identity.

In 2011, a young plant from a secondary habitat in a road embankment above the cliffs was transferred to the Botanical Garden of the University of Vienna (thereafter HBV) for cultivation and karyological investigations. Chromosome numbers were determined applying the standard Feulgen staining technique as described in Weiss-Schneeweiss et al. (2009). Actively growing root tip meristems were pre-treated with a 0.002M aqueous solution of 8-hydroxyquinoline for 2.5 h at room temperature and 2.5 h at 4 °C, fixed in ethanol:acetic acid (3:1) for at least 3 h at room temperature and stored at –20 °C until use. Fixed root meristems were hydrolysed in 5N hydrochloric acid (HCl) for 30 min at room temperature, washed with tap water and stained with Schiff’s reagent (Merck, Darmstadt, Germany) in darkness for 1 h (Jang et al. 2013). Chromosome spreads were prepared by squashing stained root tip meristems in a drop of 60% acetic acid under the cover-slip and analysed using an AxioImager M2 microscope (Carl Zeiss). All images were acquired with a CCD camera and files processed using AxioVision version 4.8 (Carl Zeiss). Karyotypes were made from these images in Corel Photo-Paint X5 (Corel Corp., Ottawa, Ontario).

For morphological comparison with the new species, specimens from the Herbaria W and WU of the other *Thliphthisa* taxa were examined with respect to diacritical characters as given in Schönbeck-Temesy and Ehrendorfer (1985). For a comprehensive list of analysed herbarium material from Greece, see Suppl. material 1.

Taxonomy and nomenclature in this work follows the Flora Ionica Working Group (2016 onwards).

## ﻿Taxonomic treatment and discussion

### ﻿ Thliphthisa sapphus

Taxon classificationPlantaeGentianalesRubiaceae

Gutermann sp. nov.

73AAE8AB-E68E-5A22-A794-ED4F17509E4A

urn:lsid:ipni.org:names:77339919-1

**Figure 1.**
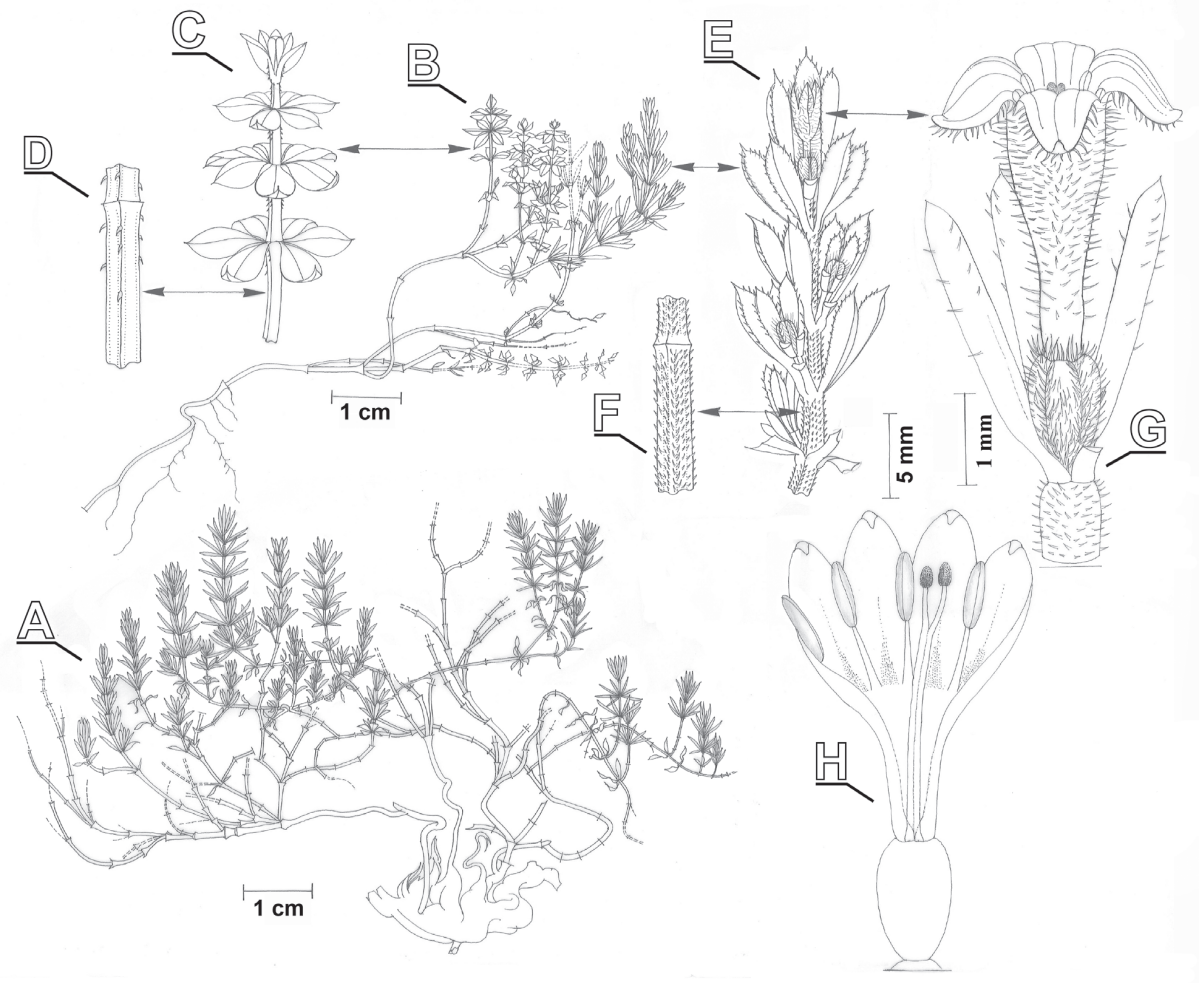
Illustration of *Thliphthisasapphus*, based on the type material **A** whole plant **B** whole plant with vegetative and flowering shoots **C** vegetative shoot **D** detail of stem of vegetative shoot **E** flowering shoot **F** detail of stem of flowering shoot **G** flower **H** dissected flower (drawings by A. Kästner).

**Figure 2.**
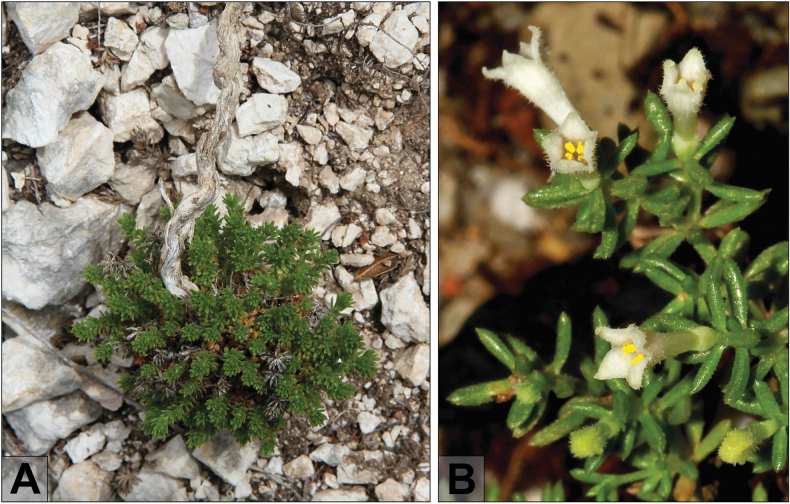
Habit and flowers of *Thliphthisasapphus***A** old individual of *Th.sapphus* with a thick lignified rootstock **B** flowers of *Th.sapphus* exhibiting the characteristic indumentum on the corolla (photographs by M. Sonnleitner).

**Figure 3.**
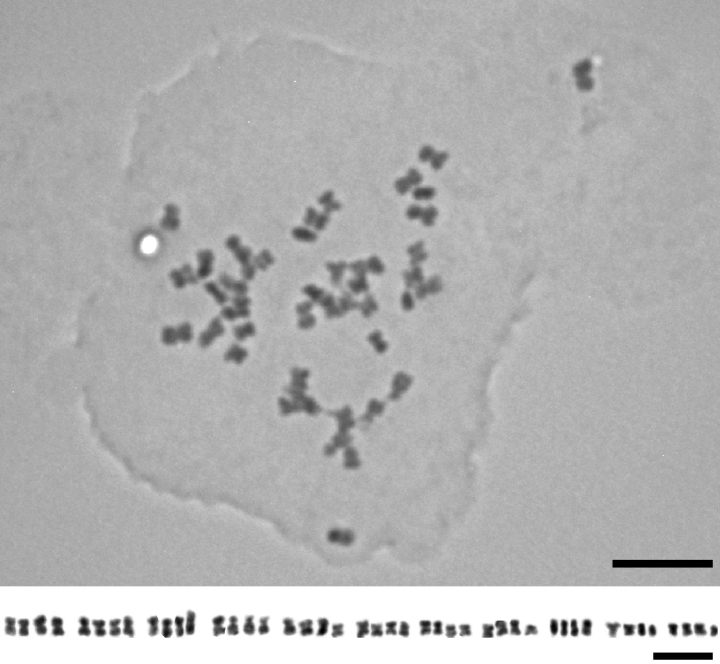
Mitotic chromosomes and karyotype of *Thliphthisasapphus* (2*n* = 4*x* = 44). Scale bar: 5 μm.

**Figure 4.**
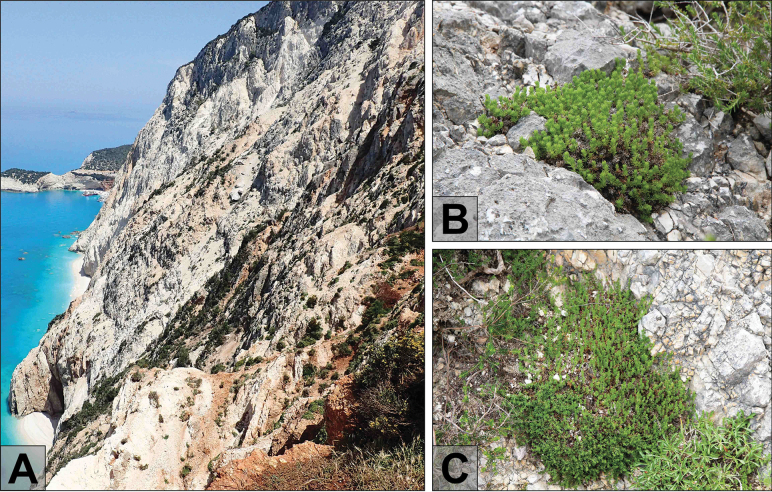
Habitat and habit of *Thliphthisasapphus***A** coastal cliffs of the Lefkátas Peninsula, the locality of *Th.sapphus* (photograph by M. Hofbauer) **B** cushion of *Th.sapphus* in limestone rock fissure (photograph by M. Sonnleitner) **C** loose cushion of *Th.sapphus* within regolith cover on limestone rocks, together with Lomelosiacrenatasubsp.dallaportae (photograph by M. Sonnleitner).

Figs 1 , 2 , 3 , 4B, C

#### Type.

Greece. Ionian Islands: Nom. Lefkada, Lefkada, Halbinsel Lefkátas, Hangkante über der Westküste WNW ober Aghios Nikólaos Níras (NNE Akr. Dhoukáto, ca. 8 km SSW Atháni), 38°35'43"N, 20°33'06"E, meerseitige Kalkfelsfluren, ca. 230 m elev., 24 May 2000, W. Gutermann et al. 35241 (holotype: WU 0154346; isotypes: ATH, K, M, LD, UPA, herb. Gutermann).

#### Description.

Generis *Thliphthisae* species nova: ***suffrutex*** nanus, laxe aut densius pulvinatus e caudice valde lignoso multicaulis; ***caulibus*** scabris, dense et aeque foliatis usque ad apicem; ***foliis*** verticillatis quaternis vel senis, leniter crassiusculis, anguste oblanceolatis brevissime apiculatis, modice setosis; ***floribus*** solitariis sessilibus; ***corollis*** hypocrateriformibus, albis, extus setosis; ***staminibus*** in medio corollae inserta, antheris luteis; ***ovariis*** ± dense setosis praesertim ad apicem versus, stylo manifeste bifido stigmatibus breviter ellipsoideis. – Chromosomatum numerus 2*n* = 44, scil. species tetraploidea. Crescit in declivibus apricis saxosis insulae Leucas Graeciae. Floret mense Maio & Junio.

Caespitose, suffruticose cushions with lignified rootstock and numerous slender ± short herbaceous shoots, up to 6 cm tall (in cultivation up to 10 cm), internodes scabrous, shorter than leaves (in cultivation somewhat longer), leaves regularly spaced, internodes with retrorse indumentum of minute setose hairs approx. 0.1 mm in length, retrose on vegetative and antrorse on flowering shoots. ***Leaves*** and leaf-like stipules in rather closely-spaced whorls of (4–)6, sessile, more or less succulent in life, linear to narrowly oblanceolate, 0.8–1(1.2) × 4–5(7) mm with short-apiculate apex, with sparsely setose margins and lower mid-ribs. ***Inflorescence*** terminal, frondose, cymes on short setose peduncles ca. 1–3 mm long, the individual cymes reduced to solitary flowers; ***flowers*** sessile, subtended by 4 setose prophylls; ***calyx*** reduced; ***corolla*** 4-merous, salverform, (crème-)white, externally ± densely setose with patent hairs 0.2–0.3 mm long, internally glabrous, the tube 2.5–3.5 mm long, the lobes ca. 1 mm long; ***anthers*** 4, yellow, glabrous, ca. 0,8 mm long, included in the tube, filaments ca. 0,8 mm long, inserted ca. 2/3 of the tube; ***style*** glabrous, bifid, the last ca. 0.5 mm free, the 2 stigmas slightly elongated to ellipsoid, included in the tube; ***ovary*** ca. 1.3 mm long, apically truncate, ± densely setose especially towards the apex, with patent hairs 0.2–0.3 mm long. Mature infructescence and **fruits** unknown.

#### Affinities of *Thliphthisasapphus*.

Due to the chamaephytic habit, with leaves (and leaf-like stipules) in rather closely-spaced whorls of 4–6, the presence of prophylls at the base of the sessile 4-merous flowers and the apically truncate ovary, *Thliphthisasapphus* belongs to the subclade of the "*Sherardia* clade" (Manen et al. 1994; Natali et al. 1995), which is now classified as genus *Thliphthisa* (Del Guacchio and Caputo 2020). Within this group defined by Schönbeck-Temesy and Ehrendorfer (1985), the new species belongs to a group of chamaephytic taxa with presumably plesiomorphic flower structures, sharing well-developed corolla tubes and slightly elongated (not globose) stigmata. This group comprises *Th.crassula* (Greuter & Zaffran) P.Caputo & Del Guacchio, *Th.tournefortii* (Sieber) P.Caputo & Del Guacchio (group A of the former study), *Th.baenitzii* (Heldr. ex Boiss.) P.Caputo & Del Guacchio and *Th.muscosa* (Boiss. & Heldr.) P.Caputo & Del Guacchio (group B). Other species with similar flower morphology are xeromorphic subshrubs deviating by their broomrape-like appearance.

*Thliphthisasapphus* is most similar to *Th.baenitzii*, but is readily distinguished by mericarps and corolla being ± densely setose (vs. glabrous), by its longer corolla of approximately 3.5–4.5 mm (vs. 2.5–3.5 mm in *Th.baenitzii*; Ehrendorfer and Krendl (1976), own measurements; the corolla length of 3–4 mm as given in Schönbeck-Temesy and Ehrendorfer (1985) is erroneous) and, in particular, by its tetraploid chromosome number that so far is unique within *Thliphthisa* (see below). *Thliphthisamuscosa* is also similar, but is well differentiated from both by its laxer growth, longer and ± erect flowering stems and distinctly longer, linear leaves.

#### Specimens examined.

Additional gatherings (paratypes). Same locality as holotype: 10 November 1994, W. Gutermann et al. 28787 (WU 0154344, herb. Gutermann 28787); 14 July 1996: E. Hörandl 7766 (ATHU, WU 0154343, herb. Gutermann 80000); 26 April 2011: W. Gutermann et al. 39920 (WU 0154345, herb. Gutermann 39920).

#### Distribution and biogeographic considerations.

To our present knowledge *Thliphthisasapphus* is restricted to a small area around the type locality on the western edge of the Lefkátas Peninsula in south-western Lefkada. Restricted distribution is common in the genus *Thliphthisa* which generally comprises eastern Mediterranean oreophytes distributed east of the Adriatic Sea and reaching the Alborz Mts. of northern Iran (see maps in Schönbeck-Temesy and Ehrendorfer (1985): figs 9–12). Only three species with more derived flower characters (shortened corolla tubes and globose stigmata) occupy larger areas, notably *Th.chlorantha* (Boiss. & Heldr.) P.Caputo & Del Guacchio and *Th.rupestris* (Vis.) P.Caputo & Del Guacchio (NW Balkan mountain ranges), and especially *Th.purpurea* (L.) P.Caputo & Del Guacchio (Balkan Peninsula and Apennines to southern and south-western Alps). Apart from three Iranian species, all other species are scattered around the east Mediterranean Basin and have a more restricted distribution. This is especially true for the taxa that are morphologically most similar to *Th.sapphus* and that are known only from single mountain stocks: *Th.baenitzii* on Mt. Patéras and *Th.muscosa* on Mt. Olympus.

These three Hellenic steno-endemics form the north-western counterpart of the South Aegean *Th.tournefortii* and its local Cretan relative *Th.crassula* (the latter two constitute group A of Schönbeck-Temesy and Ehrendorfer (1985)). *Thliphthisatournefortii* is a species of peculiar distribution (map in Runemark (1969: 115); Greuter (1971)) that can be associated chorologically with a “mesogaeic” element and, consequently, with a differentiation dating back to the Miocene (Greuter 1970). The same may be assumed for the above-mentioned more northern taxa *Th.baenitzii*, *Th.muscosa* and *Th.sapphus* sharing the more primitive flower structures with *Th.tournefortii*. The connections between these “sisters” are bridged geographically by another steno-endemic, still undescribed *Thliphthisa* species (Gutermann et al., in prep.) of Aetolia that, in habit and foliage, approaches the Cretan *Th.crassula*. Thus, *Th.sapphus* constitutes the westernmost outpost of this cluster of Greek steno-endemics. They underline the importance of cliffs as refugial habitats for poorly-competitive old chamaephytes (informally referred to as “Old ladies of the rocks”) through the times (as e.g. discussed in Davis (1951) and Snogerup (1971)).

#### Etymology.

The epithet commemorates Sappho, the most famous poetess of the Classical Hellenic Era and refers to the traditional (though non-historical) believe she suicidally threw herself from the white cliffs of Lefkátas because of unrequited love for beautiful young Phaon. The epithet is in genitive spelling. The genitive in classical Latin also is "Sapphūs", as testified in the poem delivered as "incerti avctoris epistvla Sapphvs ad Phaonem" in Ovid’s Epistolae Heroidum no. 15.

#### Karyology.

The chromosome number of *Thliphthisasapphus* is here determined as 2*n* = 4*x* = 44. The base chromosome number of *x* = 11 was found in all previously investigated representatives of the genus *Thliphthisa* [i.e. all sub *Asperula*: *Th.chlorantha*, *Th.muscosa* (Faure and Pietrera 1969), *Th.purpurea*, *Th.rupestris* (Moore 1982), *Th.crassula*, *Th.tournefortii* (Montmollin 1986), *Th.baenitzii* (Constantinidis et al. 1997), *Th.antalyensis* (Ehrend.) P.Caputo & Del Guacchio, *Th.brevifolia* (Vent.) P.Caputo & Del Guacchio, *Th.pseudochlorantha* (Ehrend.) P.Caputo & Del Guacchio, *Th.serotina* (Boiss. & Heldr.) P.Caputo & Del Guacchio (Minareci and Yildiz 2011)], as well as in most Rubieae (Kiehn and Berger 2020, 2023). However, except for *Th.sapphus*, all the investigated taxa of *Thliphthisa* are diploid. The representative karyotype of *Th.sapphus* is mostly composed of metacentric and submetacentric chromosomes (Fig. 3), as reported in other Turkish *Thliphthisa* (formally Asperulasect.Thliphthisa) taxa (Minareci and Yildiz 2011).

#### Phenology and growth.

Flowering of *Thliphthisasapphus* commences in May and, by early July, mostly withering or wilted corollas were observed. At this time, mericarps were not developed even in wilted flowers, but some were conspicuously and unusually swollen and infected by parasites, possibly gall midges (Diptera, Cecidomyiidae) as found parasitising on other Rubiaceae taxa in the Mediterranean area (e.g. Simova-Tošić et al. (2000); Skuhravá and Skuhravý (2016); Skuhravá et al. 2020). The plant cultivated in the HBV never developed fertile fruits, thus suggesting self-infertility. However, we have also failed to find fully-developed fruits *in situ*. The propagation rate of *Th.sapphus* seems to be very low, as nearly all cushions observed within this area are old plants with a thick lignified rootstock and taproot (of 1 cm ∅ and more) anchored in the rock fissures (Fig. 2A).

#### Ecology and phytosociology.

It dwells on steep to nearly vertical limestone cliffs facing the sea and is, thus, exposed to westerly winds, either in fissures in solid rock or within a thin regolith cover (Fig. 4). However, these habitats at elevations of 200–230 m a.s.l. border the slightly elevated plateau of the Peninsula and show no marked salt influence. Whereas large parts of the plateau are dominated by low *Quercuscoccifera* L. garrigue, areas with shallower and rockier soils, especially along the upper slope of the coastal cliffs, are covered by sclerophyllous phrygana vegetation with *Anthyllishermanniae* L., *Ericamultiflora* L., Juniperusphoeniceasubsp.turbinata (Guss.) Nyman or *Thymbracapitata* (L.) Cav. Depending on the distribution of narrow rock ledges, erosion fissures/crevices and compact rock, the phrygana forms an intricate mosaic with sparse chasmophytic vegetation. As demonstrated by the vegetation relevés (Table 1), *Thliphthisasapphus* is accompanied by another long-lived and cushion-forming chasmophyte Lomelosiacrenatasubsp.dallaportae (Boiss.) Greuter & Burdet showing scattered occurrences along the western coasts of the southern Ionian Islands (Flora Ionica Working Group 2016 onwards) and having also few isolated populations in Apulia (Italy) on Monte Gargano (Wagensommer 2012). Usually, together with *Plocamacalabrica* (L.f.) M.Backlund & Thulin (Rubiaceae), they form a particular plant community covering the rock fissures.

**Table 1.** *Thliphthisasapphus*-*Lomelosietumdallaportae* Mucina: vegetation relevés were performed using a modified Braun-Blanquet cover-abundance scale (see Barkman et al. (1964) for details) (10 November 1994: 1–4; 16 May 1996: 5–11).

Running number	1	2	3	4	5	6	7	8	9	10	11
Species richness	12	8	10	6	18	20	29	11	6	14	17
Altitude (m)	235	230	230	230	230	230	230	230	230	230	230
Aspect	W	W	W	NW	SSW	SW	WNW	SW	NW	NW	NNW
Inclination (°)	60–70	60–90	80	70–90	60	40	40–50	0–90	80	70	40
Sampled area (m^2^)	3	3	9	6	8	9	4	7	6	8	9
Projected cover of vegetation (%)	25	10	40	30	30	60	50	80	35	40	40
**Association character-taxa**											
Lomelosiacrenatasubsp.dallaportae (Boiss.) Greuter & Burdet	2b	2a	3	2b	2b	2a	3	3	3	3	2b
*Thliphthisasapphus* Gutermann	2m	2a	2a	1	2a	2a	2b	3	2a	2a	2a
*Plocamacalabrica* (L.f.) M.Backlund & Thulin	+	2m	+	+	2a	1	2a	1	1	+	·
**Asplenietea trichomanis**											
Brassicacreticasubsp.aegaea (Heldr. & Hal.) Snogerup, M.A.Gust. & Bothmer	·	·	·	·	·	r	r	·	·	·	·
*Ptilostemonchamaepeuce* (L.) Less.; (juv.)	·	·	·	·	·	r	·	·	·	·	·
*Stachysionica* Halácsy	·	·	·	·	·	·	·	+	·	·	·
*Campanulaversicolor* Sm.	·	·	·	·	·	·	r	·	·	·	·
Melicaminutasubsp.minuta L.	·	·	·	·	·	·	+	·	·	·	+
**Cisto-Micromerietea**											
*Ericamultiflora* L.	+	2a	2b	2a	2a	2b	+	·	·	·	·
*Thymbracapitata* (L.) Cav.	+	·	·	·	r	+	+	·	·	+	r
*Anthyllishermanniae* L.	·	·	·	2a	+	2b	+	·	·	+	1
*Lotushirsutus* L.	+	·	·	·	+	+	1	·	·	·	·
Carlinacorymbosasubsp.graeca (Heldr. & Sartori) Nyman	·	·	·	·	r	·	·	1	·	+	1
*Carexillegitima* Ces.	·	·	·	·	·	1	2a	·	·	·	·
*Convolvuluselegantissimus* Mill.	·	·	·	·	·	·	+	·	·	+	+
Micromeriagraecasubsp.graeca (L.) Benth. ex Rchb.	·	·	+	·	·	·	·	·	·	·	·
*Cistuscreticus* L.	·	·	·	·	·	·	1	·	·	·	·
*Cistussalviifolius* L.	·	·	·	·	·	·	·	·	·	·	1
*Fumanathymifolia* (L.) Webb	·	·	·	·	·	·	+	·	·	·	·
*Cytisuslanigerus* DC.; (juv.)	·	·	·	·	·	+	·	·	·	·	·
**Quercetea ilicis**											
*Smilaxaspera* L.	+	·	·	·	·	·	+	·	·	·	·
*Loniceraimplexa* Aiton	·	·	·	·	·	·	+	·	·	·	·
*Pistacialentiscus* L.; (juv.)	·	·	·	·	·	+	·	·	·	·	·
*Phillyrealatifolia* L.; (juv.)	·	·	·	·	·	·	·	·	·	·	·
*Quercuscoccifera* L.; (juv.)	·	·	·	·	·	·	r	·	·	·	·
*Cotinuscoggygria* Scop.; (juv.)	·	·	·	·	·	+	·	·	·	·	·
**Stipo-Trachynietea**											
*Catapodiumrigidum* (L.) C.E.Hubb.	+	+	·	·	r	r	+	1	·		+
*Lysimachialinum-stellatum* L.	·	·	·	·	r	·	r	·	·	·	·
Arenarialeptocladossubsp.leptoclados (Rchb.) Guss.	·	·	·	·	+	·	·	·	·	·	+
*Hypochaerisachyrophorus* L.	·	·	·	·	r	·	·	·	·	·	·
*Festucaincurva* (Gouan) Gutermann	·	·	·	·	·	·	+	·	·	·	·
*Valantiamuralis* L.	·	·	·	·	·	·	+	·	·	·	·
*Anthemischia* L.	·	·	·	·	·	·	r	·	·	·	·
*Biscutelladidyma* L.	·	·	·	·	·	·	·	·	·	·	+
**Chenopodietea**											
*Loliumrigidum* Gaudin	·	·	·	·	·	·	+	·	+	·	·
*Bromusmadritensis* L.	·	·	·	·	·	·	·	·	·	·	+
*Valerianellamicrocarpa* Loisel.	·	·	·	·	·	·	+	·	·	·	·
*Trifoliumphysodes* Steven ex M.Bieb.	·	·	·	·	·	·	·	·	·	+	·
*Geraniumpurpureum* Vill.	·	·	+	·	·	·	·	·	·	·	·
*Avenabarbata* Pott ex Link	·	·	·	·	·	·	·	·	·	·	+
**Lygeo-Stipetea**											
*Brachypodiumretusum* (Pers.) P.Beauv.	·	·	·	·	1	2b	·	2a	+	2a	·
*Leontodongraecus* Boiss. & Heldr.	+	r	r	·	·	r	+	+	·	+	+
Dactylisglomeratasubsp.hispanica (Roth) Nyman	·	·	·	·	·	·	+	+	·	·	·
Helichrysumstoechassubsp.barrelieri (Ten.) Nyman	·	·	·	·	+	+	·	·	·	·	·
*Reichardiapicroides* (L.) Roth	+	·	·	·	·	·	+	·	·	·	·
**Other taxa**											
*Lotuscytisoides* L.	·	·	+	+	·	+	·	2a	·	1	1
*Iberiscarnosa* Willd.	+	r	·	·	r	·	·	·	·	·	·
Petrosedumcf.ochroleucum (Chaix) Niederle	+	1	·	·	2m	r	·	·	·	+	·
*Taraxacum* [§ *Scariosa*] sp.	·	·	·	·	·	·	·	+	r	·	·
Festucajeanpertiisubsp.achaica (Markgr.-Dann.) Markgr.-Dann.	·	·	·	·	·	·	·	·	·	1	r
*Bromusfasciculatus* C.Presl	·	·	·	·	·	·	1	·	·	·	·
*Salviaverbenaca* L.; (juv.)	·	·	·	·	·	·	·	+	·	·	·
*Trifoliumcampestre* Schreb.	·	·	·	·	·	·	+	·	·	·	·
*Medicagolupulina* L.	·	·	·	·	·	·	·	·	·	+	·
*Bupleurumtrichopodum* Boiss. & Spruner	+	·	·	·	·	·	·	·	·	·	·
*Crucianellalatifolia* L.	·	·	·	·	+	·	·	·	·	·	·
*Linumcorymbulosum* Rchb.	+	·	·	·	·	·	·	·	·	·	·
*Scorpiurussubvillosus* L.	·	·	·	·	·	·	·	·	·	·	+
**Mosses and lichens**											
*Tortula* sp.	1	·	·	·	·	·	·	·	·	·	·
Unknown moss	·	·	+	·	·	·	·	·	·	·	·
*Lecanora* sp.	·	·	2m	·	·	·	·	·	·	·	·

We here describe this community as a new association, the *Thliphthisasapphus*-*Lomelosietumdallaportae* Mucina *ass. nova hoc loco* (holotype: relevé 9 in Table 1) and classify this association tentatively in the *Caromultiflori*-*Aurinionmegalocarpae* Terzi et D’Amico 2008, an alliance of thermo-mesomediterranean chasmophytic vegetation of limestone rock crevices of the south-eastern Italian Adriatic (Apulia) and Ionian coasts (see Mucina et al. (2016)). The syntaxonomic unit floristically and ecologically most similar is the *Scabiosetumdallaportae*Bianco et al. 1988 (see Bianco et al. (1988): table 4; Di Pietro and Wagensommer (2008: 198), table 3), an association described from Apulia, yet classified within the *Asperuliongarganicae*Bianco et al. 1988 (for the syntaxonomic discussion of the *Caromultiflori*-*Aurinionmegalocarpae* and the *Asperuliongarganicae*, see Di Pietro and Wagensommer (2008)).

#### Conservation status.

In the area directly accessible to us, we counted about two dozen mature individuals of *Thliphthisasapphus*. Within the range of sight, further cushions were identified in the nearly vertical cliff slopes below. The size of this single known population can be estimated at roughly 100 individuals, although more populations may exist in the inaccessible cliffs continuously extending north and south of the known population. In 2011, a few individuals were also found in *Erica* L. phrygana above the slopes and established on the gravelly banks along a close-by road above the primary cliff habitat. The occurrence on competition-free road banks suggests some potential of the species to colonise open anthropogenic sites, for which an occurrence in unstable gravelly habitats may have been a pre-adaptation.

Due to the restricted known geographic range, the number of individuals may be drastically reduced by natural hazards, such as land or rock slides. The population is not exposed to immediate synanthropic threats, except for goats that graze even the steepest rock declivities such as the less precipitous slopes. Due to the small number of recorded individuals distributed within a range much less than 1 km^2^, the species falls into IUCN criterion D. Taking into account the possible threats mentioned above, it should be assessed as “Vulnerable” D2 (see IUCN (2022)). At national or regional scale, it classifies as a range-restricted endemic (as “r” in Dimopoulos et al. (2013)) and needs to be included in the “Red Data Lists” for Greece as “Endangered”. Further monitoring of the population is certainly needed and conservation measures, such as reduction of grazing, should be taken for the subpopulation in the accessible area at the plateau above the ridge.

### ﻿Key to the Greek species (and subspecies) of the genus *Thliphthisa* (modified from Ehrendorfer and Krendl (1976); Schönbeck-Temesy and Ehrendorfer (1985)).

**Table:** 

1	Stigma globose	**2**
2	Corolla cup-shaped to rotate; main axis of synflorescence slender, lower branches elongate	***Th.chlorantha***
2*	Corolla infundibuliform; main axis of synflorescence robust, branches rather short	**3**
3	Corolla lobes acute; caespitose mountain plant usually < 20 cm in height	**4**
4	Corolla orange, 1–2 mm in length, corolla lobes outside with short stiff hairs; bracts frondose, longer than pedicels	***Th.pusilla***
4*	Corolla yellowish, 0.7–1 mm in length, corolla lobes outside glabrous; bracts small, shorter than pedicels	***Th.saxicola***
3*	Corolla lobes apiculate; plants of lower elevations, stems usually 20–45 cm in height	**5 *Th.purpurea***
5	Leaves gradually acuminate, arranged in whorls of 7–10, margins distinctly revolute; corolla purple, apex of corolla lobes distinctly apiculate	**Th.purpureasubsp.purpurea**
5*	Leaves abruptly acuminate, arranged in whorls of 6–8, margins plane or only slightly revolute; corolla yellowish-green or purplish, apex of corolla lobes weakly apiculate	**Th.purpureasubsp.acuminata**
1*	Stigma ellipsoid to elongated	**6**
6	All leaves elliptic to broadly ovate or suborbiculate, apex rounded	**7**
7	Plant green (not glaucous), stems prostrate; leaves slightly succulent, hirsute; synflorescence short-cylindrical in outline; corolla approx. 3 mm in length	***Th.crassula***
7*	Plant glaucous, stems erect (up to 30(35) cm); leaves coriaceous, glabrous; synflorescence pyramidal in outline; corolla 3–5 mm in length	***Th.tournefortii***
6*	At least upper leaves linear to (ob)lanceolate, apex acute	**8**
8	Subshrubs; leaves caducous	**9**
9	Leaves 12–20 mm in length; corolla 6–7 mm in length, lobes ending in filiform appendices 1.5–1.7 mm in length; mericarps 2–3 mm in length	***Th.elonea***
9*	Leaves up to 10 mm in length; corolla 2–5(6) mm in length, lobes ending in short-triangular appendices 0.3–0.5 mm in length; mericarps 1–1.5 mm in length	**10**
10	Corolla 2–3.5 mm in length, reddish or yellowish; styles 2, separate, each approx. 1 mm in length	***Th.rigida***
10*	Corolla 3.5–5(6) mm in length, greenish-yellowish or brownish-green; style 1, furcate only towards the apex, approx. 3–3.5 mm in length	***Th.brevifolia***
8*	Plant herbaceous or woody only at the base; leaves persistent	**11**
11	Plants with erect flowering stems up to 16(20) cm in height; leaves narrowly lanceolate to linear, 0.8–1.5 × 9–12 mm, long acuminate	***Th.muscosa***
11*	Plants forming low, caespitose cushions 2–6(10) cm in height; leaves broadly elliptical to (ob)lanceolate, 0.8–2 × 4–7 mm, minutely acuminate	**12**
12	Corolla 3.5–4.5 mm in length, corolla lobes outside as well as the mericarps densely setose; leaves uniformly oblanceolate	***Th.sapphus***
12*	Corolla 2.5–3.5 mm in length, corolla lobes outside glabrous to sparsely hairy, mericarps glabrous; lower leaves elliptical, upper ones broadly lanceolate	***Th.baenitzii***

## Supplementary Material

XML Treatment for Thliphthisa sapphus

## ﻿Additional information

### Author ORCIDs

Walter Gutermann^†^https://orcid.org/0000-0002-9201-6872

Tae-Soo Jang https://orcid.org/0000-0002-5527-1137

David Prehsler https://orcid.org/0000-0003-1821-8051

Dieter Reich https://orcid.org/0000-0003-0784-0048

Andreas Berger https://orcid.org/0000-0003-3902-3827

Christian Gilli https://orcid.org/0000-0002-6679-4654

Markus Hofbauer https://orcid.org/0000-0003-4235-7940

Margarita Lachmayer https://orcid.org/0000-0001-8369-9037

Ruth Sander https://orcid.org/0009-0003-9873-8889

Michaela Sonnleitner https://orcid.org/0000-0002-2026-8229

Ladislav Mucina https://orcid.org/0000-0003-0317-8886

### Data availability

All of the data that support the findings of this study are available in the main text or Supplementary Information.

## Supplementary materials

Supplementary material 1 Specimens of Greek representatives of genus *Thliphthisa* examined Data type docx https://binary.pensoft.net/file/1022511This dataset is made available under the Open Database License (http://opendatacommons.org/licenses/odbl/1.0/). The Open Database License (ODbL) is a license agreement intended to allow users to freely share, modify, and use this Dataset while maintaining this same freedom for others, provided that the original source and author(s) are credited.Walter Gutermann, Tae-Soo Jang, Arndt Kästner, David Prehsler, Dieter Reich, Andreas Berger, Ruth Flatscher, Christian Gilli, Markus Hofbauer, Margarita Lachmayer, Ruth Sander, Michaela Sonnleitner, Ladislav Mucina
