# Supplementary material for: ﻿Thliphthisasapphus (Rubiaceae, Rubieae), a new species from Lefkada (Ionian Islands, Greece) and its ecological position
Source: PhytoKeys. 2024 Apr 9;241:65–79. doi: 10.3897/phytokeys.241.119144 (PMC11024513; doi:10.3897/phytokeys.241.119144)
Supplement: Supplementary material 1 — Specimens of Greek representatives of genus Thliphthisa examined [file phytokeys-241-065_article-119144__-s001.docx]

**Specimens of Greek representatives of genus *Thliphthisa* examined.**

1) *Thliphthisa baenitzii* (Heldr. ex Boiss.) P. Caputo & Del Guacchio: **GREECE. Attica:** In rupium fissuris regionis abietinae montis "Pateras" Atticae occidentalis, alt. 3500'–4000', 8 June 1876, C. Bartholomathos De Heldreich Herbar. Florae Hellenicae 75 (syntypes [of *Asperula baenitzii* Heldr. ex Boiss.]: [WU 0127109](https://wu.jacq.org/WU0127109), [WU 0127610](https://wu.jacq.org/WU0127610), [WU 0282858](https://wu.jacq.org/WU0282858)); In mte. Pateras, May 1876, T. Pichler Pl. Graeciae exsicc. ([WU 0282859](https://wu.jacq.org/WU0282859), [WU 0282860](https://wu.jacq.org/WU0282860)); In monte "Pateras", 6 June 1911, B. Tuntas Herbarium Tuntasium 1300 ([WU 0127111](https://wu.jacq.org/WU0127111)); In regione abietina montis Parnethis, 8 July 1911, B. Tuntas Pl. exsicc. Florae hellenicae 1299 ([WU 0127110](https://wu.jacq.org/WU0127110)).

2) *Thliphthisa brevifolia* (Vent.) P. Caputo & Del Guacchio: **GREECE. South Aegean:** [Kastelorizo, Καστελλόριζο]. Prope coenobium Ajia Triadha, 16 April 1974, W. Greuter 11909 ([WU 0154297](https://wu.jacq.org/WU0154297)). **TURKEY. Antalya:** An der Straße von Manavgat, 29 May 1962, F. Ehrendorfer 62-1 ([WU 0154299](https://wu.jacq.org/WU0154299)); C3. An der Straße von Manavgat, 48 km S Akseki, 29 May 1962, F. Ehrendorfer 62-1/56-14 ([WU 0154296](https://wu.jacq.org/WU0154296)); C3. 2 km w Antalya, 27 May 1962, F. Ehrendorfer 62-1/45-40 ([WU 0154300](https://wu.jacq.org/WU0154300)). **Muğla:** (C2): Meeresbucht Ölü Deniz (= Totes Meer) (8 km Luftlinie, 15 Straßen-km S von Fethiye). Küste der die Bucht meerwärts abgrenzende[n] Landzunge, 25 March 1978, G. Fischer & M.A. Fischer 12 ([WU 0154296](https://wu.jacq.org/WU0154296)).

3) *Thliphthisa chlorantha* (Boiss. & Heldr.) P. Caputo & Del Guacchio: **ALBANIA. Gjirokastër:** M. Trebešinj [Mount Trebeshinë, Maja e Trebeshinës] supra Damesi [Damës] distr. Tepelen, 19 July 1894, A. Baldacci Iter albanicum alterum 25 ([WU 0154308](https://wu.jacq.org/WU0154308)). Vlorë: In humidis schistaceis ad Veruise distr. Kuč, 26 July 1892, A. Baldacci Iter albanicum alterum 139 ([WU 0154310](https://wu.jacq.org/WU0154310)). **GREECE. Achaia:** In m. Omplo [Omplos, Ομπλός] pr. Patras, 6 July 1899, T.H.H. von Heldreich ([WU 0154304](https://wu.jacq.org/WU0154304)). **Ioannina:** M. Kerovouni [Xerovouni, Ξεροβούνι] supra Kalentza [Kalentzi, Καλέντζι] distr. Janina, 21 August 1895, A. Baldacci Iter albanicum (epiroticum) III 153 ([WU 0154307](https://wu.jacq.org/WU0154307)); Epirus boreale-orientalis. In rupibus calcareis ad radices mt. Peristeri [Oros Lakmos] prope pagum Kalarrytae. Alt. 1000 m, 14 July 1893, E. von Halácsy ([WU 0154309](https://wu.jacq.org/WU0154309)). **Karditsa:** Agrapha (Dolopia veterum): in declivibus montis Ghavéllu Pindi supra Sermeniko, alt. 4500'–5500', 30 June 1885, T.H.H. von Heldreich ([WU 0154314](https://wu.jacq.org/WU0154314)); Thessalia. In saxosis montis "Gionscala" prope "Sermenico", August 1896, P.E.E. Sintenis Herbarium Normale (I. Dörfler) 3940 ([WU 0154306](https://wu.jacq.org/WU0154306)); Sermeniko: in mte. Gionscala, 10 August 1896, P.E.E. Sintenis Iter thessalicum 1151 ([WU 0154303](https://wu.jacq.org/WU0154303)). **Phocis:** In m. Korax Aetoliae adjectae in reg. super. ad rupes. Pyrgos [Pirgos, Πύργος], 26 July 1879, T.H.H. von Heldreich ([WU 0127589](https://wu.jacq.org/WU0127589)); Aetolia adjecta: in rupibus regionis abietinae montium Vardussia [Vardousia, Βαρδούσια] supra Vustinitza, 1 August 1896, B. Tuntas & C. Leonis Herbarium Graecum normale (T.H.H. Heldreich) 1339 ([WU 0154305](https://wu.jacq.org/WU0154305)). Trikala: Pindus (Aspropotamitikos hodie): in valle superiori Acheloi (Aspropotamo hod.), alt. 4000'–4500'; prope Kotura, 22 July 1885, T.H.H. von Heldreich ([WU 0154312](https://wu.jacq.org/WU0154312)); Pindus: in regione inferiori montis Babá prope Klinovo, alt. 3000'–4000', 23 July 1885, T.H.H. von Heldreich ([WU 0154311](https://wu.jacq.org/WU0154311)).

4) *Thliphthisa crassula* (Greuter & Zaffran) P. Caputo & Del Guacchio: **GREECE. Lasithi:** Ost-Kreta, Phrygana, Cap Mavros. NO von Erempolis, 1 October 1976, H. Meusel ([WU 0154289](https://wu.jacq.org/WU0154289)).

5) *Thliphthisa elonea* (Iatroú & T. Georgiadis) P. Caputo & Del Guacchio: **GREECE. Lakonia:** Ep. Epidhavros Limiras: Mt. Korakia N of the village Richea, alt. 500–700 m, 8 June 1995, G. Iatrou et al. OPTIMA Iter VII 1906 ([W 0196069](https://w.jacq.org/W0196069)).

6) *Thliphthisa muscosa* (Boiss. & Heldr.) P. Caputo & Del Guacchio: **GREECE. [Larissa vel Pieria]:** In pinetis reg. mediae m. Olympi Thessali, July 1851, T.H.H. von Heldreich (syntype [of *Asperula muscoa* Boiss. & Heldr.]: [WU 0127112](https://wu.jacq.org/WU0127112)); In pinetis reg. mediae m. Olympi Thessali, 21 July 1851, T.H.H. von Heldreich 2458 (possible type [of *A. muscosa* Boiss. & Heldr.]: [WU 0154294](https://wu.jacq.org/WU0154294)); Mt. Olympus Thessalus, 26 July 1905, L. Adamović Iter graeco-turcicum 486 ([WU 0127114](https://wu.jacq.org/WU0127114), [WU 0154293](https://wu.jacq.org/WU0154293)); In sylvis umbrosis montis Olympi Thessaliae, 16 July 1857, T.G. Orphanides Flora graeca exsiccata 562 ([WU 0127113](https://wu.jacq.org/WU0127113)).

7) *Thliphthisa purpurea* (L.) P. Caputo & Del Guacchio: **ALBANIA. Gjirokastër:** In umbrosis silvarum m. Trebešinj [Mount Trebeshinë, Maja e Trebeshinës] distr. Tepelen, 19 July 1894, A. Baldacci Iter albanicum alterum 26 ([WU 0154324](https://wu.jacq.org/WU0154324)). **BULGARIA. Blagoevgrad:** In dumosis mts. Jel-tepe [Vihren, Pirin mountains] Perin-dag, June 1909, M. Dimonie ([WU 0154320](https://wu.jacq.org/WU0154320)). **GREECE. Chalkidiki:** Macedonia: in reg. super mtis Athos [Άθως], June 1908, M. Dimonie ([WU 0127116](https://wu.jacq.org/WU0127116)); Hagion Oros mtis Athos [Άθως], June 1908, M. Dimonie ([WU 0154316](https://wu.jacq.org/WU0154316), [WU 0154317](https://wu.jacq.org/WU0154317)); In pinetis Hagion Oros cönobium Prodrom, May 1909, M. Dimonie ([WU 0154318](https://wu.jacq.org/WU0154318)); Peninsula Hagion Oros. Mt. Athos, bei Panagia, 22 June 1891, P.E.E. Sintenis & J.F.N. Bornmüller Iter turcicum 857 ([WU 0127115](https://wu.jacq.org/WU0127115)). **Ioannina:** In praeruptis m. Konitza sub Papingon (Vradeton), 14 July 1896, A. Baldacci Iter albanicum (epiroticum) IV 239 ([WU 0154325](https://wu.jacq.org/WU0154325)); Montes Pindus: in declivibus siccis prope Metsovo, 28 July 1956, K.H. Rechinger 18659 ([WU 0154313](https://wu.jacq.org/WU0154313)). **Karditsa:** Sermeniko: in mte. Gionscala, 10 August 1896, P.E.E. Sintenis Iter thessalicum 1152 ([WU 0154322](https://wu.jacq.org/WU0154322)). **[Larissa vel Pieria]:** Macedonia: mt. Olympus Thessalus, 26 July 1905, L. Adamović Iter graeco-turcicum 476 ([WU 0154319](https://wu.jacq.org/WU0154319)). **Pieria:** In atois umbrosis Olympi Thessaliae supra Hagios Dionysios, 28 July 1857, T.G. Orphanides Flora graeca exsiccata 555 ([WU 0154315](https://wu.jacq.org/WU0154315)); Macedonia. Olympos thessal.: in silvis prope Hagios Dionysios, 12 September 1889, P.E.E. Sintenis Iter orientale 1889 1856 ([WU 0154321](https://wu.jacq.org/WU0154321)).

8) *Thliphthisa pusilla* (Bory & Chaub.) J. Krieg., Del Guacchio &. P. Caputo: **GREECE. [Arkadia vel Lakonia]:** In m. Malevô Laconiae, s.d., C. Leonis ([WU 0154332](https://wu.jacq.org/WU0154332)). **Arkadia:** Laconia orientalis (i. e. Cynuria): in rupinis m. Malevô (Parnon veter.) prope cacumen, alt. 6000', 5 July 1896, C. Leonis Herbarium Graecum Normale (T.H.H. Heldreich) 1337 ([WU 154330](https://wu.jacq.org/WU0154330)). **Lakonia:** In rupestribus mt. Taygetos supra Anavryti, 25 May 1902, C. Leonis Plantae exsiccatae florae graecae (E. Halácsy) 106 ([WU 0154331](https://wu.jacq.org/WU0154331)). **Messinia:** Laconia boreo-occidentalis: in rupestribus m. Selitza, prope Kalamata, K.H. Zahn Herbarium Graecum Normale (T.H.H. Heldreich) 1337b ([WU 0154329](https://wu.jacq.org/WU0154329)).

9) *Thliphthisa rigida* (Sm.) P. Caputo & Del Guacchio: **GREECE. Chania:** Kreta, Nom. Chania, oberer Abschnitt der Samaria-Schlucht, 1 July 1979, H. Meusel 22-2 ([WU 0154295](https://wu.jacq.org/WU0154295)). **Lasithi:** In saxos. ad torrentes. Mirabello, s.d., F.W. Sieber & F. Kohaut ([WU 0127611](https://wu.jacq.org/WU0127611)). **Rethymno:** Kreta. Distrikt Hag. Vasilis. An Felsen der Schlucht "Khordaliotikon - Pharangi", 19 June 1904, I. Dörfler Iter Creticum 1904 (I. Dörfler) 636 ([WU 0103019](https://wu.jacq.org/WU0103019)).

10) *Thliphthisa saxicola* (Ehrend.) P. Caputo & Del Guacchi: **GREECE. Arkadia:** Large vertical rocks facing NW, 820–860 m s.m., 3 km ENE of the village of Agiorgitika (c. 15 km E of Tripolis), 12 July 1966, A. Strid 23386 (isotype [of *Asperula saxicola* Ehrend.]: [WU 0154334](https://wu.jacq.org/WU0154334)).

11) *Thliphthisa tournefortii* (Sieber ex Spreng.) P. Caputo & Del Guacchio: **GREECE. Heraklion:** Candia [Heraklion, Ηράκλειο], 1817, F.W. Sieber (syntypes [of *Asperula tournefortii* Sieber ex Spring.]: [WU 0127107](https://wu.jacq.org/WU0127107), [WU 0127609](https://wu.jacq.org/WU0127609)); In rupestribus supra Kani Kastello distr. Temenos, 2 July 1899, A. Baldacci Iter creticum alterum 26bis ([WU 0154292](https://wu.jacq.org/WU0154292)); In rupestribus m. Joukta distr. Temenos, 6 June 1899, A. Baldacci Iter creticum alterum 26ter ([WU 0154291](https://wu.jacq.org/WU0154291)); In rupestribus ins. Dhia distr. Megalokastron, 30 May 1899, A. Baldacci Iter creticum alterum 26 ([WU 0154290](https://wu.jacq.org/WU0154290), [WU 0127108](https://wu.jacq.org/WU0127108)); Insula Dia, Ormos Panagias, in fissuris rupium calc., 27 Jule 1973, K.H. Rechinger Iter Graecum XVIII., 1973 45809 ([WU 0103020](https://wu.jacq.org/WU0103020)).
